# Saponin Inhibits Hepatitis C Virus Propagation by Up-regulating Suppressor of Cytokine Signaling 2

**DOI:** 10.1371/journal.pone.0039366

**Published:** 2012-06-20

**Authors:** Jihye Lee, Seri Lim, Sang-Min Kang, Saehong Min, Kidong Son, Han Sol Lee, Eun Mee Park, Huong T. T. Ngo, Huong T. L. Tran, Yun-Sook Lim, Soon B. Hwang

**Affiliations:** National Research Laboratory of Hepatitis C Virus, Ilsong Institute of Life Science, Hallym University, Anyang, Korea; Yonsei University, Republic of Korea

## Abstract

Saponins are a group of naturally occurring plant glycosides which possess a wide range of pharmacological properties, including anti-tumorigenic and antiviral activities. To investigate whether saponin has anti-hepatitis C virus (HCV) activity, we examined the effect of saponin on HCV replication. HCV replication was efficiently inhibited at a concentration of 10 µg/ml of saponin in cell culture grown HCV (HCVcc)-infected cells. Inhibitory effect of saponin on HCV replication was verified by quantitative real-time PCR, reporter assay, and immunoblot analysis. In addition, saponin potentiated IFN-α-induced anti-HCV activity. Moreover, saponin exerted antiviral activity even in IFN-α resistant mutant HCVcc-infected cells. To investigate how cellular genes were regulated by saponin, we performed microarray analysis using HCVcc-infected cells. We demonstrated that suppressor of cytokine signaling 2 (SOCS2) protein level was distinctively increased by saponin, which in turn resulted in inhibition of HCV replication. We further showed that silencing of SOCS2 resurrected HCV replication and overexpression of SOCS2 suppressed HCV replication. These data imply that saponin inhibits HCV replication via SOCS2 signaling pathway. These findings suggest that saponin may be a potent therapeutic agent for HCV patients.

## Introduction

Hepatitis C virus (HCV) is a major cause for chronic liver disease leading to liver cirrhosis and hepatocellular carcinoma (HCC) [1]. More than 170 million people worldwide are infected with HCV. HCV is an enveloped, positive-sense RNA virus classified in the *Hepacivirus* genus within the *Flaviviridae* family. HCV has been classified into six major genotypes and numerous subtypes [2]–[4]. HCV genome encodes a single polyprotein precursor of more than 3,010 amino acids, which is cleaved into structural (core, E1, and E2) and nonstructural (p7, NS2 to NS5B) proteins by host and viral proteases at the endoplasmic reticulum. A vaccine is not yet available and the only licensed therapy for patients infected with HCV is a combination of the pegylated interferon (IFN)-α and ribavirin. The therapy with these agents is associated with various adverse effects and achieves a sustained virological response (SVR) with significant differences among genotypes [5]. Although two inhibitors of HCV protease, boceprevir (Victrelis™) and telaprevir (Incivek™), are recently approved by the Food and Drug Administration, these drugs are only effective in combination with peginterferon alpha and ribavirin. Furthermore, small molecule inhibitors of HCV RNA polymerase are in clinical trial stages, the error prone nature of the viral RNA polymerase leads to rapid emergence of viral-resistant mutations to these therapeutic candidates [6]. Therefore, herbal medicine could be an alternative approach to control HCV propagation. It has been reported that catechin, glycyrrhizin, silymarin and phytosterol showed efficacy in therapy of chronic hepatitis [7]. Proanthocyandin, purified from blueberry leaves, inhibited viral replication in HCV-infected patients [8]. Recently, it has been reported that the laccase, an extract from oyster mushroom, suppressed HCV entry into peripheral blood cells and hepatoma cells [9]. Furthermore, Hussein *et al.* reported that medicinal plant extracts from *Acacia nilotica, Boswellia carterii, Embelia schimperi, Piper cubeba, Quercus infectoria, Trachyspermum ammi,* and *Syzygium aromaticum* significantly inhibited HCV protease activity *in vitro* [10].

Saponins are mainly produced by plants but also by lower marine animals and some bacteria [11], [12]. Saponins possess a wide range of pharmacological properties, including anti-carcinogenic, anti-inflammatory, and anti-viral activities. HIV-1 replication was inhibited by triterpenoid sapogenin oleanolic acid, which probably decreased the HIV-1 protease activity. In addition, some triterpenoidal saponins have been reported to show anti-herpes simplex virus type 1 (HSV-1) activity [13], [14]. In the present study, we investigated whether saponin exhibited anti-HCV activity in HCVcc-infected cells. We performed microarray analysis using HCVcc-infected cells to examine which cellular gene expressions were modulated by saponin. Of deregulated genes, suppressor of cytokine signaling 2 (SOCS2) level was increased ∼6 times by saponin treatment as compared to the untreated control. We showed that saponin inhibited HCV propagation by up-regulating SOCS2 protein level. Silencing of SOCS2 thereby restored HCV propagation in HCV-infected cells. These data suggest that saponin may be a potential candidate for anti-HCV therapeutic agent.

## Materials and Methods

### Cell Culture and Transfection

All cell lines were cultured in Dulbecco’s modified Eagle’s medium (DMEM) supplemented with 10% fetal bovine serum (FBS), and 100 units/ml of penicillin-streptomycin in 5% CO_2_ at 37°C. Huh7 cells harboring HCV subgenomic replicon and IFN-cured cells were grown as previously reported [15]. For the transfection experiment, ∼5×10^5^ cells plated on 60-mm dishes were transfected with plasmid DNA using either Lipofectamine (Invitrogen) or polyethyleneimine reagent (Sigma) according to the manufacturer’s instructions. cDNA encoding SOCS2 was prepared from Huh7.5 cells and cloned into p3XFLAG-CMV10 (Sigma) expression vector. The forward primer of SOCS2 was 5′-ATG AAT TCA ATG ACC CTG CGG TGC CTT-3′ and reverse primer was 5′-TAT GGA TCC TTA TAC CTG GAA TTT ATA TTC-3′.

### Preparation of Infectious Virus

The infectious HCVcc was generated as described previously [16]. Briefly, HCV RNAs (Jc1, adaptive mutant, IFN-resistant mutant, JFH1-luc) were generated using T7 RiboMAX™ Express Large Scale RNA Productive System (Promega) according to the manufacturer’s protocols. Approximately, 7.5×10^6^ Huh7.5 cells (a gift from Dr. Charles M. Rice, Rockefeller University) were resuspended in 400 µl of cytomix solution containing 2 mM ATP and 5 mM glutathione, mixed with 10 µg of *in vitro* transcribed HCV RNA in a 4-mm gap cuvette, and then electroporated at 300 V and 975 µF using a GenePulser II electroporator (Bio-Rad). Cells were gently transferred to complete medium (low glucose DMEM containing 10% FBS, 100 units/mL penicillin, 100 µg/mL streptomycin, 2 mM L-glutamine, 1 mM NEAA and 10 mM HEPES) and plated on a 150-mm dish. At 24 h later, the medium was replaced with the fresh complete medium to remove cell debris. The culture medium was collected at 4 days after electroporation, filtered through a 0.45-mm filter unit, and kept as a virus stock. The culture supernatant harvested from cells transfected with Jc1/GNN mutant RNA was prepared as described above and used as a mock infection.

### Cytotoxicity Assay

Host cell viability at various concentrations of saponin (CALBIOCHEM, Germany) was determined using EZ-CyTox cell viability assay kit (DAEILLAB, Korea) according to the manufacturer’s protocol.

### Immunoblot Analysis

Cells were harvested and lysed in cell lysis buffer containing 50 mM Tris-HCl (pH 7.5), 150 mM NaCl, 1% Nonidet P-40, 1 mM EDTA, 0.25% sodium deoxycholate, 1 mM Na_3_VO_4_, 1 mM sodium fluoride, 1 mM phenylmethylsulfonyl fluoride, 1 mM β-glycerophosphate, and protease inhibitor mixture (Roche) for 15 min on ice. The cell lysates were further centrifuged at 15,000×g for 15 min at 4°C. The protein concentration was determined by the Bradford assay (Bio-Rad). Equal amounts of proteins were subjected to either 8% or 15% SDS-PAGE and electrotransferred to a nitrocellulose membrane. The membrane was blocked in PBS containing 5% nonfat dry milk for 1 h and then incubated 2 h at room temperature with one of following primary antibodies: rabbit anti-NS3 and anti-NS5A sera [17], anti-SOCS2 and SOCS3 antibodies (Epitomics), anti-STAT1 antibody (Santa Cruz), anti-p-STAT1 antibody (Cell Signaling), and anti-β actin antibody (Sigma) in Tris-buffered saline/Tween (20 mM Tris-HCl (pH 7.5), 500 mM NaCl, and 0.05% Tween 20). Following two washes in Tris-buffered saline/Tween 20, the membrane was incubated with either horseradish peroxidase-conjugated goat anti-rabbit antibody or goat anti-mouse antibody (Jackson ImmunoResearch Laboratories, West Grove, PA) in Tris-buffered saline/Tween 20 for 1 h at room temperature. Proteins were detected using an ECL kit (Elpis Biotech, Korea).

### Quantification of HCV RNA

RNAs were isolated from HCVcc-infected cells, cell culture medium, or replicon cells by using RiboEx™ Total RNA (GeneAll Biotechnology, Korea) according to the manufacturer’s instructions. Purified viral RNA was used as a template to synthesize cDNA using the TOPscript™ cDNA synthesis kit (Enzynomics, Korea). cDNA was amplified with HCV genotype 2a-specific primers (forward, AGAGCCATAGTGGTCTGCGGAAC; reverse, CCTTTCGCAACCCAACGCTACTC), HCV genotype 1b-specific primers (forward, ATCACTCCCCTGTGAGGAACTACT; reverse, CTGGAGGCTGCACGACACTC), SOCS2-specific primers (forward, GAGCTCGGTCAGACAGGATG; reverse, AGTTGGTCCAGCTGATGTTTT), and GAPDH-specific primers (forward, CGCTCTCTGCTCCTCCTGTTC; reverse, CGCCCAATACGACCAAATCCG). All quantitative real-time PCR (qRT-PCR) experiments were performed by using an iQ5 multicolor real-time PCR detection system (Bio-Rad Laboratories, Hercules, CA) under the following conditions: 15 minutes at 95°C followed by 40 cycles of 95°C for 20 s, 55°C for 20 s, 72°C for 20 s. Seventy one cycles of 10 s, with 0.5°C temperature increments from 60 to 95°C, were used for the melting curves. Quantification of HCV RNA was normalized with GAPDH RNA as an internal control and was shown as percentage of HCV RNA.

### Reporter Assays

Huh7.5 cells were electroporated with *in vitro* transcribed JFH1-Luc RNA. At 48 h after RNA electroporation, cells were treated with increasing amounts of saponin in the absence or presence of 50 U/ml of IFN-α (Sigma-Aldrich, St. Louis, MO) for 24 h and then luciferase reporter activities were determined as described previously [16].

### Gene Silencing by siRNA

siRNAs targeting the SOCS2, the 5′UTR of HCV (positive control), and the universal negative control were purchased from Dharmacon (Lafayette, CO). siRNA transfection was performed using a Lipofectamine RNAiMax reagent (Invitrogen, Carlsbad, CA) according to the manufacturer’s instructions.

### Microarray Analysis

Huh7.5 cells were infected with Jc1 for 4 h and then either left untreated or treated with 10 µg/ml of saponin. At 24 h after saponin treatment, cellular RNAs were isolated by using RiboEx™ Total RNA (GeneAll Biotechnology, Korea) according to the manufacturer’s instructions. The extracted RNAs were labeled using a GeneAtlas™ 3′IVT Express Kit Assay (Affymetrix), and hybridized onto Affymetrix Human Genome U219 Array strip that is comprised of more than 530,000 probes covering over 36,000 transcripts and variants. Raw microarray data were processed using Partek Express software (Affymetrix Edition) to generate values representing log_2_ gene expression. The calculated fold-change value means the ratio of gene expression level in saponin-treated Jc1-infected cells as compared to the non-treated Jc1-infected cells. Cellular genes that were either 2-fold up-regulated or down-regulated by saponin in Jc1-infected cells were selected as targets for further study.

## Results

### Saponin Suppresses HCV Propagation in HCVcc-infected Cells

**Figure 1:**
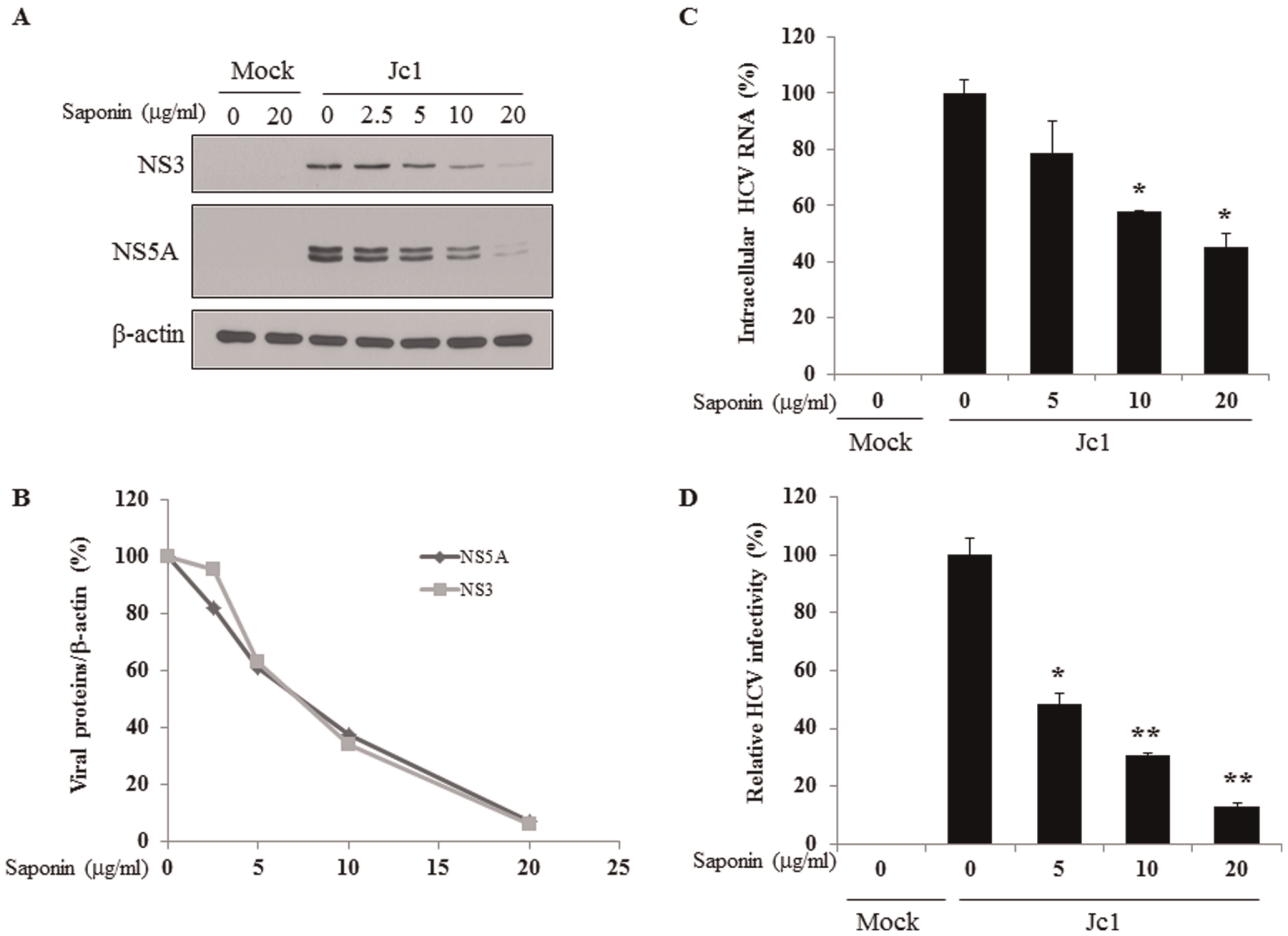
Saponin inhibits HCV propagation. (A) Saponin inhibits HCV protein expression in a dose-dependent manner. Huh7.5 cells infected with either HCV Jc1 or mock for 4 h were incubated with the selected amounts of saponin (2.5, 5, 10, and 20 µg/ml) for 24 h. Cells were harvested and then equal amounts of cell lysates were immunoblotted with anti-NS3 and NS5A antibodies, respectively. β-actin was used as a loading control for the same amount of cell lysates. (B) Relative viral protein levels in HCV-infected cells treated with the indicated dosage of saponin were normalized by β-actin. (C) Huh7.5 cells were infected with Jc1 for 4 h and then treated with the indicated amounts of saponin. At 24 h postinfection, intracellular HCV RNAs were analyzed by qRT-PCR using a primer targeting HCV 5′UTR-core. (D) Relative HCV infectivity was determined by focus-forming units (FFU) as we reported previously [16]. Error bars indicate the standard deviation. * indicates statistical significance (p<0.05) and ** indicates p<0.01 versus control.

Saponin exerts antiviral activity against HSV type I [14]. To investigate whether saponin could exhibit anti-HCV activity, we first examined the effect of saponin on HCV propagation. Huh7.5 cells were either mock-infected or Jc1-infected (genotype 2a) and then treated with various concentrations of saponin. As shown in Fig. 1A, saponin inhibited HCV protein expression in a dose-dependent manner. It was noteworthy that HCV protein expression levels were significantly inhibited with 10 µg/ml of saponin (Fig. 1A, 1B). To determine whether the inhibitory effect of saponin on HCV protein expression was caused by cytotoxicity of saponin, cellular viability was analyzed by cytotoxicity assay. We found that 20 µg/ml of saponin exerted no cytotoxicity in HCVcc-infected cells (data not shown). To further investigate whether higher dosages of saponin might increase anti-HCV activity, HCVcc-infected cells were treated with either 50 µg/ml or 100 µg/ml of saponin and then resultant HCV protein expression and cell viability were analyzed. Indeed, HCV protein levels were decreased 95% at 50 µg/ml and 99% at 100 µg/ml of saponin, respectively (Fig. S1). However, cell viability was also decreased by 20% and 30% at 50 µg/ml and 100 µg/ml of saponin, respectively. These results suggest that 10 to 20 µg/ml of saponin is the optimum concentration to inhibit HCV replication without affecting cellular growth. The half maximal inhibitory concentration (IC_50_) for saponin to inhibit HCV replication was in the range of 7–18 µg/ml of saponin in general (Fig. S2). We further showed that cellular toxicity value (CC_50_) of saponin was 165.72 µg/ml and selective index was considered significant (Fig. S2). To determine the effect of saponin on HCV RNA level, we quantified both intracellular HCV RNA level and HCV infectivity in saponin treated cells. We demonstrated that intracellular HCV RNA level was significantly reduced by 10 µg/ml of saponin. As shown in Fig. 1D, HCV infectivity was significantly suppressed by 5 µg/ml of saponin, indicating that saponin inhibits HCV propagation.

### Time Course Effect of Saponin on HCV Propagation

**Figure 2:**
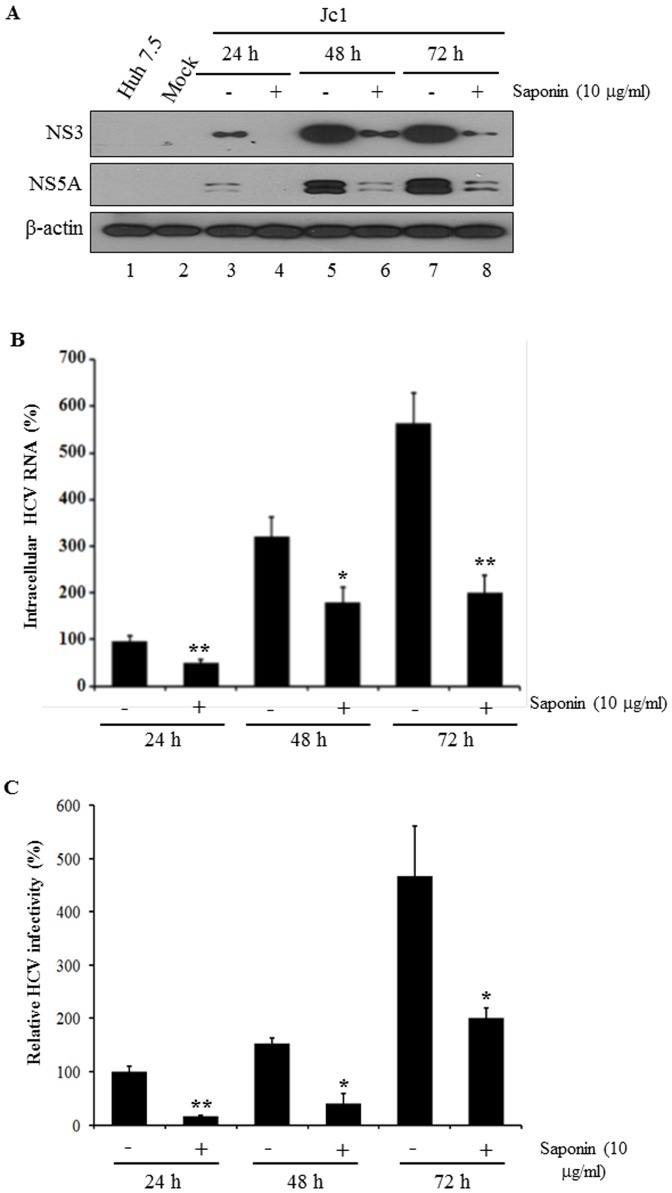
Time course effect of saponin on HCV propagation. (A) Huh7.5 cells were infected with Jc1 for 4 h and then treated with 10 µg/ml of saponin for 24 h, 48 h, and 72 h, respectively. At the indicated time points, HCV protein levels were analyzed by immunoblot analysis using anti-NS3 and NS5A antibodies, respectively. (B) Intracellular HCV RNAs were quantified by qRT-PCR at the given time intervals. (C) Relative HCV infectivity was determined by FFU as described above. The results are representative of three independent experiments. Error bars indicate the standard deviation.

To analyze the time course effect of saponin on HCV propagation, Huh7.5 cells infected with Jc1 were incubated with 10 µg/ml of saponin for 24 h, 48 h, and 72 h, respectively. As shown in Fig. 2A, HCV protein expression level was continuously increased from 24 h to 72 h in the absence of saponin. However, HCV protein expression was prominently inhibited as early as 24 h after saponin treatment and continued until 72 h. At 72 h after saponin treatment, HCV protein expression level was suppressed by ∼90% as compared to untreated control (Fig. 2A, lane 7 versus lane 8). To further analyze both intracellular HCV RNA level and HCV infectivity during the time course of experiments, the same set of experiments were performed as described in the legend to Fig. 2A and HCV RNAs were quantified by qPCR. Indeed, both intracellular HCV RNA level (Fig. 2B) and relative HCV infectivity (Fig. 2C) were significantly decreased with 10 µg/ml of saponin from 24 h to 72 h. These results confirm that saponin is a potent inhibitor for HCV replication.

### Saponin Suppresses HCV Replication in Replicon Cells

**Figure 3:**
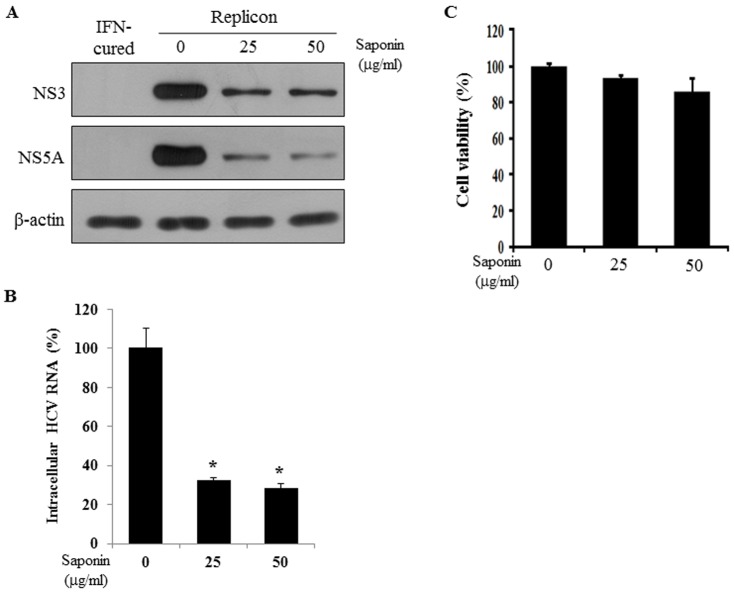
Saponin inhibits HCV replication in Huh7 cells harboring subgenomic HCV replicon. (A) HCV protein expressions were decreased by saponin. Both IFN-cured and subgenomic replicon cells were either left untreated or treated with 25 and 50 µg/ml of saponin, respectively. At 24 h after saponin treatment, total cell lysates were immunoblotted with the indicated antibodies. β-actin was used as a loading control for the same amount of cell lysates. (B) Total cellular RNAs were extracted from HCV replicon cells treated with saponin and intracellular HCV RNAs were quantified by qRT-PCR. (C) Subgenomic replicon cells were left untreated or treated with 25 and 50 µg/ml of saponin, respectively. At 24 h after saponin treatment, cell viability was determined by cytotoxicity assay.

To further investigate whether saponin could suppress viral replication in other genotype of HCV, we analyzed the effect of saponin on viral replication using HCV replicon derived from genotype 1b. Both IFN-cured and subgenomic replicon cells were either left untreated or treated with increasing amounts of saponin. At 24 h after saponin treatment, HCV protein levels were determined by immunoblot analysis. As shown in Fig. 3A, HCV protein expression levels were suppressed by saponin in a dose dependent manner. Next, we quantified intracellular HCV RNA levels by qPCR. Fig. 3B showed that intracellular HCV RNA levels were significantly decreased by 25 µg/ml of saponin. To determine whether saponin induced cytotoxicity in Huh7 cells harboring HCV replicon, cell viability was determined by cytotoxicity assay. As shown in Fig 3C, cellular toxicity was not induced by saponin. Although cell viability was slightly decreased at 50 µg/ml of saponin, this effect was insignificant. These data indicate that saponin inhibits HCV replication in both genotype 1b (subgenomic replicon cells) and genotype 2a (Jc1-infected cells). To further examine whether saponin could inhibit other virus of *Flaviviridae family*, we investigated JEV-infected BHK cells as we reported previously [16]. We found that saponin also inhibited JEV replication (data not shown). We thus speculate that saponin may not target specific HCV protein. Saponin may exert anti-HCV activity by modulating cellular signaling as described below.

### Saponin Potentiates IFN-α-induced anti-HCV Activity

**Figure 4:**
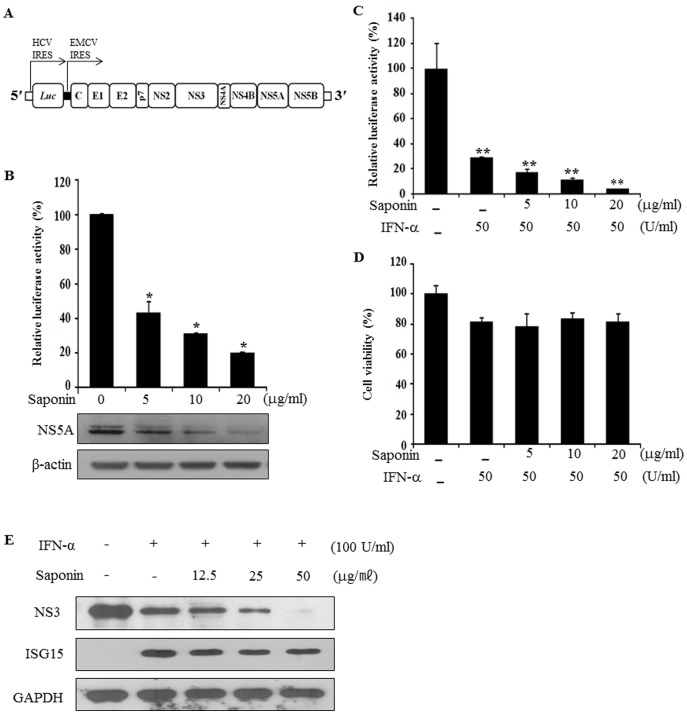
Saponin potentiates IFN-αinduced anti-HCV activity. (A) Genomic organization of JFH1-derived luciferase reporter construct (JFH1-Luc). (B) Saponin suppresses HCV reporter activity in a dose-dependent manner. Huh7.5 cells were electroporated with JFH1-Luc RNA. At 48 h after electroporation, cells were treated with increasing amounts of saponin for 24 h and then luciferase reporter activities were determined. Data represent the mean of three independent experiments. (C) Huh7.5 cells were electroporated with JFH1-Luc RNA and were treated with increasing amounts of saponin in the absence or presence of 50 U/ml of IFN-α for 24 h and then luciferase reporter activities were determined. (D) Huh7.5 cells were electroporated with JFH1-Luc RNA and then treated with both saponin and IFN-α as described in the legend to Fig. 4C and cell viability was determined by cytotoxicity assay. (E) HCV subgenomic replicon cells were treated with either IFN-α alone or in combination with IFN-α and saponin as indicated. Cell lysates harvested at 36 h postinfection were immunoblotted with anti-NS3 antibody.

To explore the possible application of saponin for combination therapy, we evaluated the anti-HCV potency of saponin in combination with IFN-α using JFH1-Luc reporter system (Fig. 4A). To verify the reporter activity first, Huh7.5 cells were electroporated with JFH1-Luc RNA, treated with increasing amounts of saponin, and then luciferase reporter activities were determined. As shown in Fig. 4B, HCV reporter activities were significantly decreased by saponin in a dose-dependent manner. Likewise, NS5A expression level was also decreased in a similar pattern (Fig. 4B, lower panel). We then examined the combinatorial effect of both IFN-α and saponin on HCV replication. Huh7.5 cells electroporated with JFH1-Luc RNA were treated with increasing amounts of saponin in the absence or presence of IFN-α for 24 h and then luciferase reporter activities were determined. Fig. 4C showed that IFN-α significantly decreased HCV reporter activity and this effect was increased by saponin in a dose-dependent manner. Importantly, HCV replication was suppressed ∼95% by co-treatment of IFN-α and saponin. Cytotoxicity data indicated that cell viability was not altered by co-treatment of IFN-α and saponin (Fig. 4D). To further analyze the effect of co-treatment of saponin and IFN-α on HCV replication, replicon cells were treated with either IFN-α alone or in combination with IFN-α and saponin as indicated. Antiviral effect of co-treatment of saponin and IFN-α on HCV replication was then analyzed by immunoblotting with anti-NS3 antibody. Fig. 4E showed that IFN-α-induced anti-HCV activity was potentiated by saponin in a dose-dependent manner.

### Saponin Inhibits IFN-α Resistant Mutant HCV Propagation

**Figure 5:**
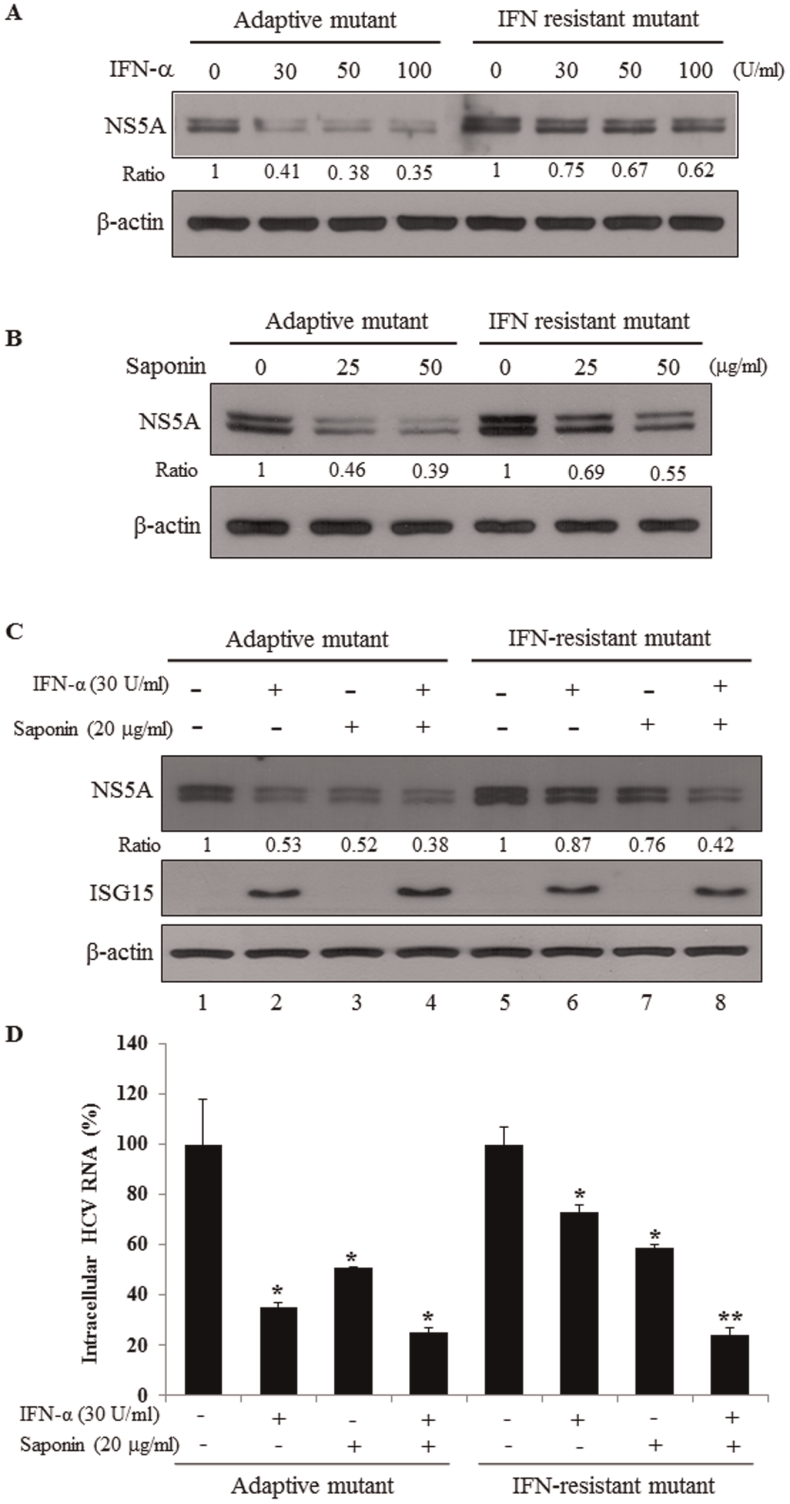
Saponin suppresses viral propagation of IFN-α resistant mutant HCV. (A) Verification of IFN-α resistance of the mutant HCV. Huh7.5 cells were infected with either adaptive mutant HCV or IFN-α resistant mutant HCV for 4 h. The cells were treated with increasing amounts of IFN-α for 24 h and then total cell lysates were immunoblotted with the indicated antibodies. β-actin was used as a loading control for the same amount of cell lysates. (B) Saponin suppresses the NS5A expression in cells infected with IFN-α resistant mutant HCV. Huh7.5 cells were infected with HCV as described in the figure legend to A and then treated with increasing amounts of saponin for 24 h. Total cell lysates were immunoblotted with the indicated antibodies. (C) Co-treatment of saponin and IFN-α synergistically inhibits HCV replication in cells infected with IFN-α resistant HCV as similar level as in cells infected with adaptive HCV. Huh7.5 cells were infected with HCV as described in the figure legend to A and then treated with either 30 U/ml of IFN-α or 20 µg/ml of saponin alone, or in combination with IFN-α and saponin as indicated. Total cell lysates were immunoblotted with the indicated antibodies. (D) Huh7.5 cells were infected with HCV and then treated with either IFN-α or saponin alone, or in combination with IFN-α and saponin as described in the figure legend to C. Intracellular HCV RNAs isolated from these cells were analyzed by qRT-PCR.

We previously established a novel HCV clone that acquired IFN-α resistant phenotype by long-term culturing HCV-infected cells in the presence of IFN-α [18]. We also generated adaptive HCV mutant in parallel by culturing HCV-infected cells in the absence of IFN-α and used as a control. Both adaptive and IFN-α resistant mutants have the same six adaptive mutations but IFN-α resistant clone has additional four amino acid substitution mutations in the C-terminal coding sequence of NS5A [18]. To verify the IFN-α resistant phenotype first, Huh7.5 cells were infected with either control adaptive mutant HCV or IFN-α resistant mutant HCV and then treated with increasing amounts of IFN-α. As shown in Fig. 5A, the IFN-α resistant mutant HCV was highly resistant to IFN-α, whereas the adaptive mutant was susceptible to IFN-α as determined by NS5A protein level. To explore the inhibitory effect of saponin on IFN-α resistant mutant, Huh7.5 cells infected with IFN-α resistant mutant HCV were treated with various concentrations of saponin. Indeed, HCV protein expression level was decreased by saponin in IFN-α resistant mutant albeit the decrease level was less than the adaptive mutant (Fig. 5B). We then investigated the effects of co-treatment of IFN-α and saponin on NS5A expression in cells infected with IFN-α resistant mutant HCV. Fig. 5C showed that co-treatment of IFN-α and saponin additively inhibited HCV protein expression in control cells infected with adaptive mutant (lane 4). Most importantly, co-treatment of IFN-α and saponin significantly inhibited NS5A protein expression even in cells infected with IFN-α resistant mutant HCV (Fig. 5C, lane 8). It was noteworthy that co-treatment of IFN-α and saponin in IFN-α-resistant mutant HCV-infected cells suppressed NS5A protein expression as much as in cells infected with adaptive mutant HCV (Fig. 5C, lane 4 versus lane 8). We further confirmed that HCV core protein expression was also similarly inhibited by co-treatment of IFN-α and saponin (data not shown), indicating that saponin exerted antiviral activity even in IFN-α resistant HCV clone. To further analyze the effect of saponin on IFN-α resistant mutant HCV more accurately, we determined intracellular HCV RNA levels by qRT-PCR. Indeed, HCV RNA levels in IFN-α-resistant mutant HCV-infected cells were similarly inhibited by co-treatment of IFN-α and saponin as in cells infected with adaptive mutant HCV (Fig. 5D). These data strongly suggest that saponin may be the promising combinatorial therapeutic agent for IFN-α non-responders.

### Saponin Suppresses HCV Replication by Regulating SOCS2

**Figure 6:**
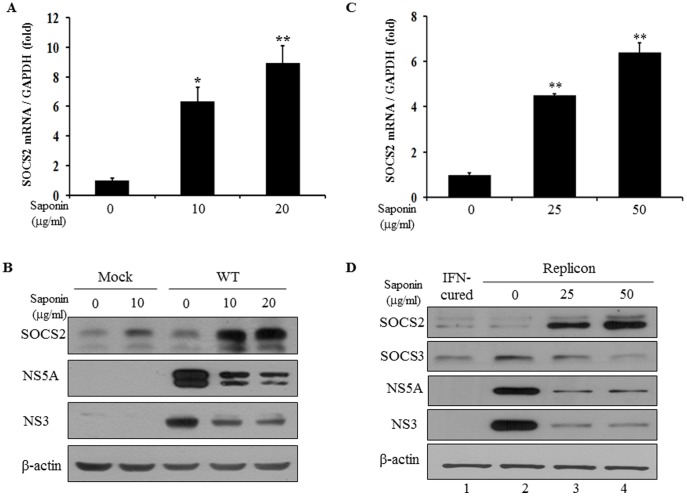
Saponin inhibits HCV replication by increasing SOCS2 expression level. (A) Huh7.5 cells were infected with Jc1 for 4 h. Cells were then either left untreated or treated with the indicated amounts of saponin for 24 h. Total cellular RNAs were extracted and then SOCS2 mRNA level was quantified by qRT-PCR. (B) Huh7.5 cells were infected with either mock or Jc1 for 4 h. The cells were then either left untreated or treated with the indicated amounts of saponin for 24 h. Equal amounts of cell lysates were immunoblotted with the indicated antibodies. (C) HCV subgenomic replicon cells were treated with increasing amounts of saponin for 24 h. Total cellular RNAs were extracted and then SOCS2 mRNA level was quantified by qRT-PCR. (D) Both IFN-cured and subgenomic replicon cells were left untreated or treated with increasing amounts of saponin for 24 h. Equal amounts of cell lysates were immunoblotted with the indicated antibodies. The protein expression levels were normalized with β-actin.

To investigate the molecular mechanism of saponin involved in anti-HCV activity, we screened cellular target genes of saponin in HCV-infected cells using microarray analysis. Huh7.5 cells were infected with HCV Jc1 and then either left untreated or treated with 10 µg/ml of saponin. Total mRNAs were isolated from each group and were subjected to microarray analysis. As shown in Table S1, 256 genes were >2-fold up-regulated and 236 genes were >2-fold down-regulated in saponin treated cells as compared to the untreated cells. Interestingly, genes involved in signal transduction pathways were predominantly increased by saponin and genes involved in energy metabolism were largely decreased by saponin in HCV-infected cells (Fig. S3). It was noteworthy that SOCS2 expression level was highly increased (6 times) in saponin-treated cells as compared to the untreated control in microarray data. Because SOCS2 has been implicated in the negative regulation of cytokine signal transduction pathway, we investigated whether SOCS2 was involved in saponin-mediated inhibition of HCV replication. Huh7.5 cells were infected with Jc1 and then treated with 10 µg/ml or 20 µg/ml of saponin. Total cellular RNAs were extracted and then SOCS2 mRNA level was quantified by qPCR. As shown in Fig. 6A, SOCS2 mRNA level was significantly increased by saponin in a dose-dependent manner in Jc1-infected cells. To further confirm whether saponin increased SOCS2 protein level, Huh7.5 cells infected with either mock or Jc1 were either left untreated or treated with the indicated amounts of saponin for 24 h. Equal amounts of cell lysates were immunoblotted with the indicated antibodies. Fig. 6B showed that SOCS2 protein expression level was increased by saponin, which in turn resulted in decrease of HCV protein expression levels. To further investigate whether saponin increased SOCS2 level in HCV subgenomic replicon cells, cells were treated with increasing amounts of saponin and then SOCS2 mRNA level was quantified by qPCR. Indeed, saponin significantly increased SOCS2 mRNA level in HCV replicon cells (Fig. 6C). We also examined SOCS2 protein level in HCV replicon cells. As shown in Fig. 6D, SOCS2 protein level was increased by saponin in a dose-dependent manner. As expected, HCV protein levels were prominently decreased by saponin. It has been reported previously that the SOCS2 induces SOCS3 degradation by forming E3 ligase complex [19]. We showed that SOCS3 level was higher in replicon cells than in IFN-cured cells (Fig. 6D, lane 1 versus lane 2). Indeed, SOCS3 level was gradually decreased as SOCS2 level was increased by saponin (Fig. 6D). Furthermore, silencing of SOCS3 with siRNA decreased HCV replication (data not shown). These data imply that saponin may suppress HCV replication via SOCS2 signal pathway.

### SOCS2 Negatively Regulates HCV Propagation

**Figure 7:**
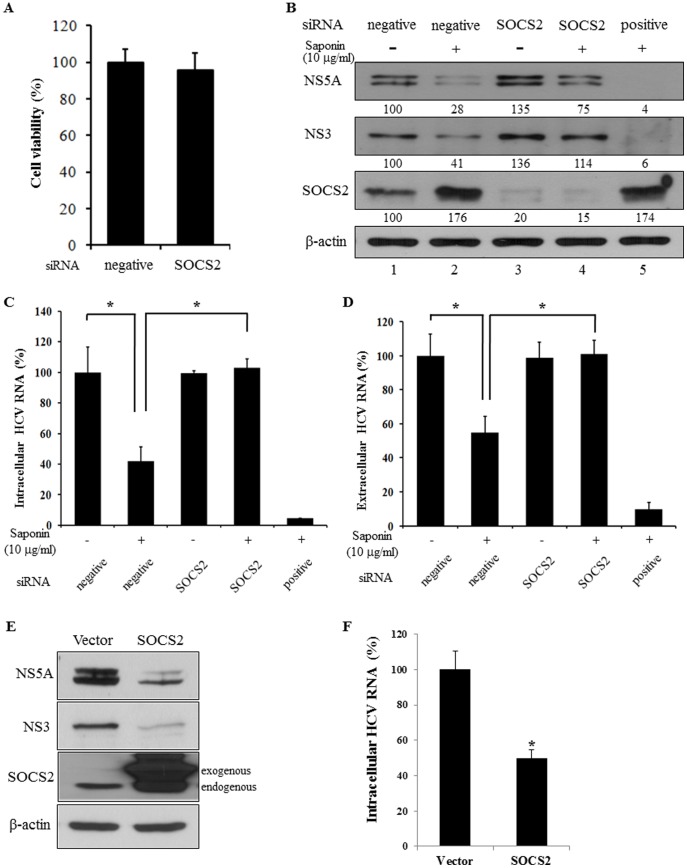
Saponin inhibits HCV propagation by regulating SOCS2 gene. (A) Huh7.5 cells were treated with either negative or SOCS2 siRNAs. At 72 h after transfection, cell viability was analyzed by cytotoxicity assay. (B-D) Silencing of SOCS2 impairs inhibitory activity of saponin on HCV propagation. Huh7.5 cells were transfected with the indicated siRNAs. At 36 h after transfection, cells were infected with HCV Jc1 for 4 h and then treated with 10 µg/ml of saponin for 24 h. Total cell lysates were immunoblotted with the indicated antibodies (B). Both intracellular HCV RNAs (C) and extracellular HCV RNAs (D) isolated from the culture supernatant were quantified by qRT-PCR. (E, F) Overexpression of SOCS2 suppresses HCV replication. Huh7 cells were transfected with either empty vector or FLAG-tagged SOCS2 expression plasmid. At 24 h after transfection, cells were infected with HCV for 4 h. Cell lysates harvested at 48 h postinfection were immunoblotted with the indicated antibodies (E). (F) Huh7 cells were treated as described in the figure legend to E. Intracellular HCV RNAs were isolated and analyzed by qRT-PCR.

To investigate whether saponin-induced SOCS2 was specifically involved in HCV propagation, HCV protein expression levels were analyzed by silencing of SOCS2 in Jc1-infected cells. Fig. 7A showed that cell viability was not affected by either negative or SOCS2 siRNA in Huh7.5 cells. We then examined the protein expression levels of both HCV and SOCS2 in cells transfected with the indicated siRNAs. As shown in Fig. 7B, HCV protein expression was significantly suppressed by saponin via up-regulating SOCS2 expression (lane 2). Indeed, silencing of SOCS2 expression enhanced HCV protein expression (Fig. 7B, lane 1 versus lane 3). However, HCV protein expressions were no longer significantly suppressed by saponin in SOCS2 knockdown cells (Fig. 7B, lane 2 versus lane 4). We further confirmed that saponin specifically inhibited HCV replication via SOCS2 in replicon cells (Fig. S4), indicating that SOCS2 played a crucial role in anti-HCV activity of saponin. To further confirm the effects of SOCS2 on HCV replication, we quantified both intracellular and extracellular HCV RNA levels by qPCR in SOCS2 knockdown cells. Saponin suppressed both intracellular HCV RNA (Fig. 7C) and extracellular HCV RNA (Fig. 7D) levels. However, saponin was unable to suppress both intracellular and extracellular HCV RNA levels in SOCS2 knockdown cells. To further investigate whether saponin could inhibit HCV replication by upregulating SCOS2, both HCV protein and intracellular HCV RNA levels were analyzed by overexpressing SOCS2 protein in HCV-infected cells. Indeed, overexpression of SOCS2 significantly suppressed both HCV protein level (Fig. 7E) and intracellular HCV RNA level (Fig. 7F). Together, these data indicate that saponin inhibits HCV propagation via SOCS2 protein and thus SOCS2 plays as a negative regulator in HCV propagation.

## Discussion

Persistent infection is the hallmark of HCV patients. HCV infection often causes chronic hepatitis, liver cirrhosis, and HCC. However, neither a protective vaccine nor an effective therapeutic agent against HCV is available yet. Only available current therapy is the combination of pegylated IFN and ribavirin. However, this therapy accompanies a high rate of side effects and SVR varies with different genotypes of HCV [6], [20]. Therefore, there is an urgent need to develop more effective therapeutic agent against HCV. Because herbal medicine could be an alternative way to control HCV propagation, we have investigated the potential anti-HCV activity of saponin. Herbal medicines or medicinal plants have been widely used to prevent and treat many diseases. The biggest advantage of herbal medicines is that those medicines are safe and abundant in nature. The naturally occurring flavonoid silymarin, an extract from *Silybum marianum*, inhibits hepatic stellate cell activation that is crucial for fibrogenesis [21]. Moreover, silymarin exerted inhibitory activities on HCV RNA replication and hepatic fibrosis in HCV patients [22]. Glycyrrhizin, a major component of Glycyrrhiza glabra extract, modified glycosylation and suppressed sialyation of hepatitis B surface antigen (HBsAg), which inhibited secretion of HBsAg [23]. The risk of the progression of hepatocellular carcinoma was also reduced 2.5-fold in chronic hepatitis C patients treated with glycyrrhizin as compared to the untreated control group [24]. Phyllanthus amarus plant exhibited therapeutic potential in chronic HBV carriers by suppressing HBV polymerase activity, HBV mRNA transcription and replication [25], [26]. Interestingly, rhizomes of the Chinese medicinal herb Rhodiola kirilowii (Regel) Maxim inhibited HCV NS3 serine protease [27].

Saponin is the major pharmacological constituent in some plants, including *Panax Ginseng* and *Platycodon grandiflorus*. Saponin has been known to exert antiviral activities in HSV and HIV [13], [14], [28]. In the present study, we demonstrated that saponin strongly inhibited HCV propagation at the levels of intracellular HCV RNA, protein expression, and extracellular HCV RNA. We showed that saponin suppressed viral replication in HCV derived from both genotype 1b (subgenomic replicon) and genotype 2a (Jc1).

Another major finding was that co-treatment of saponin and IFN-α was highly effective in suppressing HCV replication. We demonstrated that co-treatment of saponin and IFN-α suppressed HCV reporter activity for almost no detectable level at 72 h after treatment. Moreover, co-treatment of saponin and IFN-α in Huh7.5 cells infected with IFN-α resistant HCV clone suppressed HCV replication as drastically as did in IFN-α sensitive HCV. Because IFN-α therapy accompanies adverse effects in many HCV patients, co-therapy of saponin and IFN-α would minimize the adverse effects and thus will increase SVR rate in HCV patients. Collectively, these data suggest that the combination of IFN-α and saponin may be the legitimate therapy for IFN non-responders and thus could be an alternative strategy to elevate the SVR rate in other HCV genotypes.

To elucidate the molecular mechanism of saponin involved in anti-HCV activity, we screened cellular target genes of saponin in Jc1-infected cells using microarray analysis. We showed that SOCS2 gene expression was up-regulated by saponin. We further confirmed that SOCS2 expression level was also increased by saponin in HCV replicon cells. HCV protein expression level was gradually decreased as SOCS2 level was increased in cells treated with increasing amounts of saponin. SOCS2 is a member of suppressor of cytokine signaling family that includes eight members, and characterized by the presence of a SH2 domain and C-terminal SOCS box [29]. The SOCS box interacts with Elongin BC, part of an E3 ubiquitin ligase complex that degrades target proteins through the ubiquitin pathway [29]. Tannahill *et al.* reported that SOCS2 interacted with SOCS3 and degraded SOCS3 by forming E3 ligase complex using Elongin BC in SOCS2 transgenic mouse [19]. SOCS3 was induced by HCV core protein and maintained at relatively high levels in chronic hepatitis C patients [30]. SOCS family proteins are typically considered as inhibitors of IFN signaling. However, overexpression of SOCS2 inhibited HCV replication in our study. It has been reported previously that SOCS1 and SOCS3 displayed an inhibitory activity toward the activation of STAT1 in response to IFNs [31]. However, overexpression of SOCS2 had no effect on the IFN-mediated activation of STAT1 or the antiproliferative activity of IFNs. SOCS2 did not inhibit IFN-, IL-6, and OM-27, induced Jak/STAT signaling. Furthermore, SOCS2 expression enhanced the antiproliferative activity of IFNs in the presence of low concentrations of IFNs. Although SOCS2 is one of the SOCS family members, it exerts a unique function distinguishable from SOCS1 and SOCS3.

In the present study, saponin increased SOCS2 level, which in turn resulted in inhibition of HCV replication by decreasing SOCS3 level. Although the molecular mechanism of saponin-induced SOCS2 has not been fully clarified in the present study, saponin no longer suppresses HCV propagation in SOCS2 knockdown cells. These data imply that SOCS2 is specifically involved in saponin-mediated HCV suppression and thus SOCS2 is a negative regulator in HCV propagation. Taken together, our study demonstrated that saponin might be a potential therapeutic agent for HCV patients.

## Supporting Information

Table S1 **Identification of cellular genes deregulated by saponin in HCV-infected cells.** Jc1-infected cells were either left untreated or treated with saponin (10 μg/ml) for 24 h. Total mRNAs isolated from each group were subjected to microarray analysis. Either two-fold upregulated (256 genes) or downregulated (236) genes by saponin in HCV-infected cells as compared to non-treated control were selected. Data normalization and selection of fold-changed genes were performed using Gene springGX 7.3 (Agilent technology, USA). Gene ID, accession number, and functional description are provided.
(XLSX)

Figure S1 **Determination of anti-HCV activity at high concentration of saponin.** (A) Huh 7.5 cells were either mock-infected Jc1-infected for 4 h and then treated with selected amounts of saponin (10, 50, and 100 µg/ml). Cell lysates harvested at 24 h after saponin treatment were immunoblotted with anti-NS5A antibody. (B) Huh7.5 cells treated with the indicated amounts of saponin were analyzed for viability by cytotoxicity assay. Relative viral protein levels in HCV-infected cells treated with the indicated dosage of saponin are means ± standard errors for three independent experiments.
(TIF)

Figure S2 **Determination of IC_50_ and selective index in replicon cells and HCV-infected cells.** (A) To determine IC_50_ of saponin in replicon cells, Huh7 cells harboring HCV replicon were treated with various concentration of saponin for 24 h and then total RNAs were isolated to perform qRT-PCR using primer sets of HCV genotype 1b and GAPDH. To determine IC_50_ of saponin in HCV-infected cells, Huh7.5 cells were infected with HCV (Jc1, adaptive mutant JFH1, and IFN resistant mutant JFH1, respectively) for 4 h and then treated with various concentration of saponin for 24 h. Total RNAs extracted from each sample were quantified by qRT-PCR. GAPDH was used as a normalization gene for qRT-PCR analysis and data were shown as percentage of HCV RNA. For JFH1-Luc, Huh7.5 cells were electroporated with *in vitro* transcribed JFH1-Luc RNA. At 48 h after RNA electroporation, cells were treated with increasing amounts of saponin for 24 h and then luciferase reporter activities were determined. (B) IC_50_ of saponin was estimated using the line where the saponin concentration provided 50% inhibition of HCV RNA in HCV-infected cells. Selective index (SI) was determined from the ratio of CC_50_/IC_50_. CC_50_ was calculated as 165.72 µg/ml. SI>4 is considered significant.
(TIF)

Figure S3 **Functional classification of cellular genes altered by saponin in HCV-infected cells.** Jc1-infected cells were either left untreated or treated with saponin (10 µg/ml) for 24 h. Total mRNAs isolated from each group were subjected to microarray analysis. Both up-regulated and down-regulated cellular genes in saponin treated cells as compared to non-treated cells were classified by molecular functions.
(TIF)

Figure S4 **Silencing of SOCS2 impairs inhibitory activity of saponin on HCV replication in replicon cells.** Huh7 cells harboring HCV subgenomic replicon were transfected with the indicated siRNAs. At 36 h after transfection, cells were treated with 25 µg/ml of saponin for 24 h. Total cell lysates were immunoblotted with the indicated antibodies**.** β-actin was used as a loading control for the same.
(TIF)
