# Supplementary figures and images for: Saponin Inhibits Hepatitis C Virus Propagation by Up-regulating Suppressor of Cytokine Signaling 2
Source: PLoS One. 2012 Jun 20;7(6):e39366. doi: 10.1371/journal.pone.0039366 (PMC3379970; doi:10.1371/journal.pone.0039366)

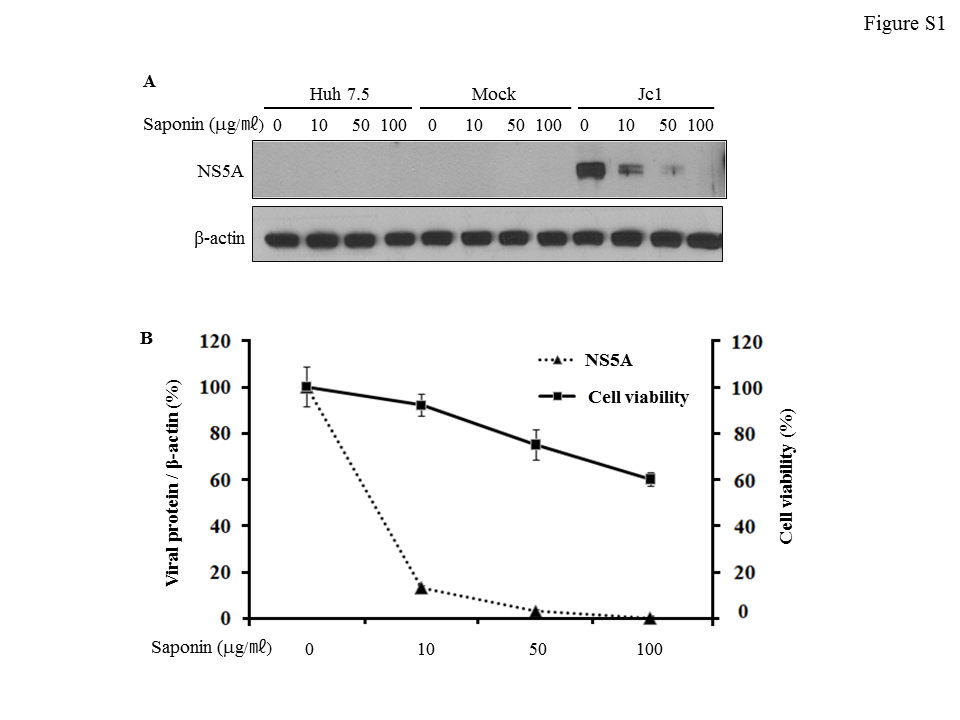

Supplement: Figure S1 — Determination of anti-HCV activity at high concentration of saponin. (A) Huh 7.5 cells were either mock-infected Jc1-infected for 4 h and then treated with selected amounts of saponin (10, 50, and 100 µg/ml). Cell lysates harvested at 24 h after saponin treatment were immunoblotted with anti-NS5A antibody. (B) Huh7.5 cells treated with the indicated amounts of saponin were analyzed for viability by cytotoxicity assay. Relative viral protein levels in HCV-infected cells treated with the indicated dosage of saponin are means ± standard errors for three independent experiments. (TIF) [file pone.0039366.s002.tif]

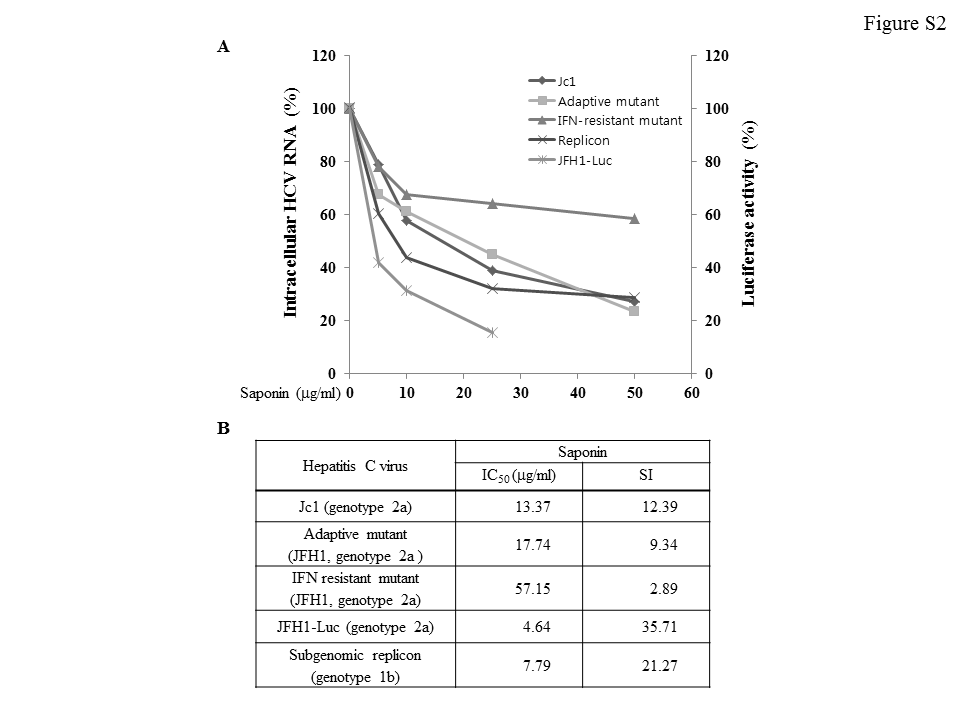

Supplement: Figure S2 — Determination of IC50 and selective index in replicon cells and HCV-infected cells. (A) To determine IC50 of saponin in replicon cells, Huh7 cells harboring HCV replicon were treated with various concentration of saponin for 24 h and then total RNAs were isolated to perform qRT-PCR using primer sets of HCV genotype 1b and GAPDH. To determine IC50 of saponin in HCV-infected cells, Huh7.5 cells were infected with HCV (Jc1, adaptive mutant JFH1, and IFN resistant mutant JFH1, respectively) for 4 h and then treated with various concentration of saponin for 24 h. Total RNAs extracted from each sample were quantified by qRT-PCR. GAPDH was used as a normalization gene for qRT-PCR analysis and data were shown as percentage of HCV RNA. For JFH1-Luc, Huh7.5 cells were electroporated with in vitro transcribed JFH1-Luc RNA. At 48 h after RNA electroporation, cells were treated with increasing amounts of saponin for 24 h and then luciferase reporter activities were determined. (B) IC50 of saponin was estimated using the line where the saponin concentration provided 50% inhibition of HCV RNA in HCV-infected cells. Selective index (SI) was determined from the ratio of CC50/IC50. CC50 was calculated as 165.72 µg/ml. SI>4 is considered significant. (TIF) [file pone.0039366.s003.tif]

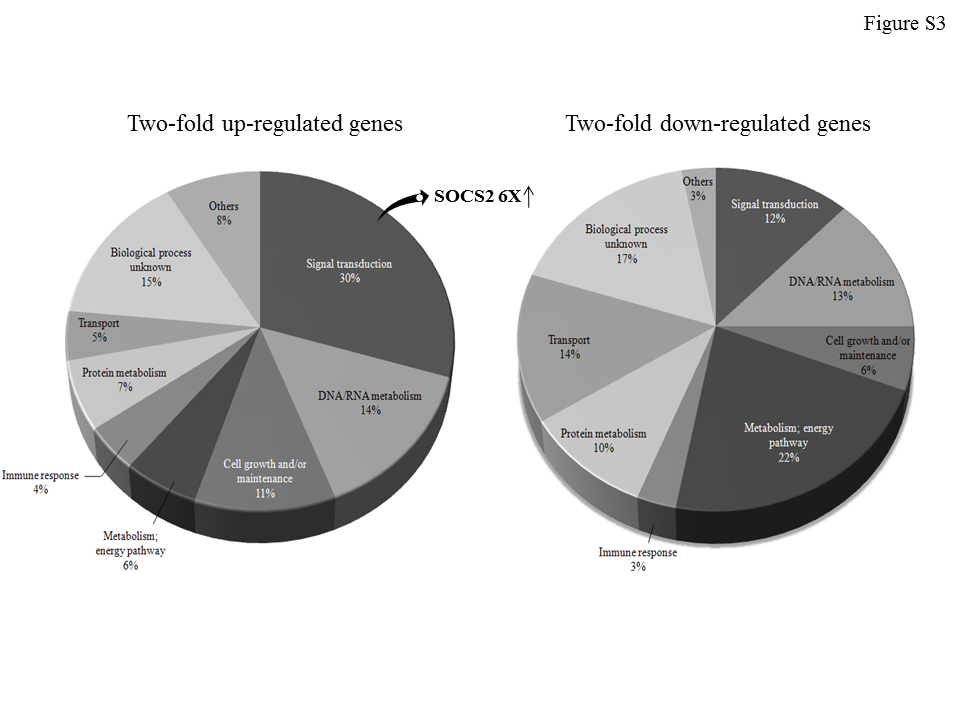

Supplement: Figure S3 — Functional classification of cellular genes altered by saponin in HCV-infected cells. Jc1-infected cells were either left untreated or treated with saponin (10 µg/ml) for 24 h. Total mRNAs isolated from each group were subjected to microarray analysis. Both up-regulated and down-regulated cellular genes in saponin treated cells as compared to non-treated cells were classified by molecular functions. (TIF) [file pone.0039366.s004.tif]

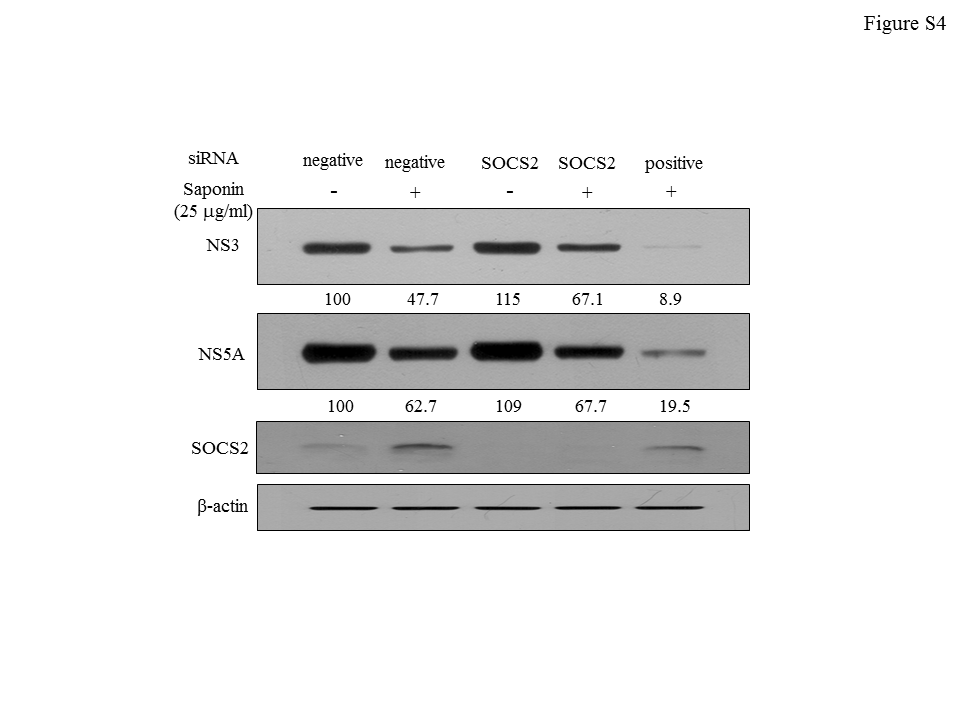

Supplement: Figure S4 — Silencing of SOCS2 impairs inhibitory activity of saponin on HCV replication in replicon cells. Huh7 cells harboring HCV subgenomic replicon were transfected with the indicated siRNAs. At 36 h after transfection, cells were treated with 25 µg/ml of saponin for 24 h. Total cell lysates were immunoblotted with the indicated antibodies. β-actin was used as a loading control for the same. (TIF) [file pone.0039366.s005.tif]
